# The developmental trajectory of attentional orienting to socio-biological cues

**DOI:** 10.1007/s00221-016-4627-3

**Published:** 2016-04-09

**Authors:** Nicola Jean Gregory, Frouke Hermens, Rebecca Facey, Timothy L. Hodgson

**Affiliations:** Department of Psychology, Faculty of Science and Technology, Bournemouth University, Poole, Dorset UK; School of Psychology, University of Lincoln, Brayford Pool, Lincoln, UK; School of Psychology, College of Life and Environmental Science, University of Exeter, Exeter, UK

**Keywords:** Attention, Gaze direction, Prefrontal cortex, Saccades, Infants

## Abstract

It has been proposed that the orienting of attention in the same direction as another’s point of gaze relies on innate brain mechanisms which are present from birth, but direct evidence relating to the influence of eye gaze cues on attentional orienting in young children is limited. In two experiments, 137 children aged 3–10 years old performed an adapted pro-saccade task with centrally presented uninformative eye gaze, finger pointing and arrow pre-cues which were either congruent or incongruent with the direction of target presentations. When the central cue overlapped with presentation of the peripheral target (Experiment 1), children up to 5 years old had difficulty disengaging fixation from central fixation in order to saccade to the target. This effect was found to be particularly marked for eye gaze cues. When central cues were extinguished simultaneously with peripheral target onset (Experiment 2), this effect was greatly reduced. In both experiments finger pointing cues (image of pointing index finger presented at fixation) exerted a strong influence on saccade reaction time to the peripheral stimulus for the youngest group of children (<5 years). Overall the results suggest that although young children are strongly engaged by centrally presented eye gaze cues, the directional influence of such cues on overt attentional orienting is only present in older children, meaning that the effect is unlikely to be dependent upon an innate brain module. Instead, the results are consistent with the existence of stimulus–response associations which develop with age and environmental experience.

## Introduction

Briefly displaying a picture of a face or eyes looking to the left or right has been shown to facilitate an observer’s attention and saccadic eye movements in the direction which the actor’s eyes are looking. This eye gaze cuing effect is found even when participants are instructed to ignore the direction in which the actor’s eyes point and the cues are uninformative of the likely direction in which objects of interest are to appear (Friesen and Kingstone 1998; Driver et al. 1999; Koval et al. 2005; Kuhn and Benson 2007; Kuhn et al. 2009; Kuhn and Kingstone 2009; Gregory and Hodgson 2012).

It has been suggested that the gaze cueing effect is important for our understanding of the development of mechanisms underpinning social interaction, theory of mind and “mind-reading” abilities in humans (Baron-Cohen 1994) and that “reflexive” orienting in response to gaze direction cues constitutes evidence for the existence of an innate, hard-wired eye gaze direction detector module within the brain (Baron-Cohen 1995). But if such an innate module exists, it would be expected that orienting of attention in response to eye gaze cues would be found from a very young age.

It is well established that babies start to orient attention spontaneously in the direction that adults look at around the end of the first year of life (Scaife and Bruner 1975; Butterworth and Jarrett 1991; Morissette et al. 1995; D’Entremont et al. 1997; Carpenter et al. 1998). However, evidence for the existence of rapid attentional orienting responses to eye gaze cues under the age of 1 year is less conclusive. Hood et al. (1998) had babies around 3 months of age view digitised faces which at first appeared to blink and then to avert their gaze to the right or left. Babies were found to make more saccades towards a peripheral target object in the direction of the observed eye gaze shift than in the other direction. Farroni et al. (2004) showed a similar effect in 2–5-day-old neonates, but found it to be dependent upon the presence of apparent motion in the cue. Cueing of attention was only found when the eyes appeared to move either from being closed or from gazing centrally. As motion cues are capable of producing a shift of attention in the direction of movement, regardless of the composition of the moving stimuli (Abrams and Christ 2003), gaze cueing effects in neonates could simply be attributed to an innate orienting response to motion rather than eye gaze direction. Farroni et al. (2000) also demonstrated a similar dependency of eye gaze cues on apparent motion in 4–5-month-olds. When the face itself was laterally shifted, leaving the pupils in the same position, the infants’ attention was oriented in the direction of the moving face, not in the direction that the actor’s pupils were gazing.

In older children (3–5 years of age), Ristic and colleagues (2002) documented larger gaze cueing effects in children compared to adults. Similarly, Neath et al. (2013) reported a gaze orienting effect in 7-year-old children which decreased in magnitude with increasing age. In a saccadic response task, Kuhn et al. (2011) demonstrated larger gaze cueing effects in 10-year-old children than in adults. These authors proposed that the development of inhibitory cognitive control mechanisms may be responsible for an attenuated gaze cue-orienting effect with increasing age (also see Kuhn et al. 2015). This may reflect the development of inhibitory control structures within the prefrontal cortex including the frontal and supplementary eye fields, dorsolateral and ventrolateral prefrontal cortex (Paus et al. 1999; Huttenlocher 2002; DeSouza et al. 2003; Kramer et al. 2005; Luna et al. 2008). It is known for example that anti-saccade error rates in children decrease systematically with increasing age (Bucci and Seassau 2012; Kramer et al. 2005; Klein and Foerster 2001).

In summary, evidence for gaze cueing effects in babies and young children only partially supports the existence of an innate eye gaze direction module in humans. Effects in neonates may be explained by apparent motion rather than gaze cueing. Strong gaze cueing effects only appear in older children and then decline with age into adulthood in a manner consistent with the development of executive/inhibitory control processes and structures within the frontal cerebral cortex. But if evidence for an innate gaze cueing module is lacking, what mechanism explains the effect of gaze and other socio-biological cues in directing attention in older children and adults?

An alternative perspective is that responses initiated by eye gaze and other socio-biological cues are not hard-wired in the traditional sense, but are acquired via repeated pairing of stimuli in the environment with orienting of attention, i.e. learned stimulus–response (SR) associations. Consistent with this idea, non-biological arrow stimuli produce very similar cueing effects (Galfano et al. 2012; Tipples 2002, 2008; Ristic et al. 2002; Quadflieg et al. 2004; Kuhn and Benson 2007; Kuhn and Kingstone 2009) although no authors to our knowledge have claimed that humans have a dedicated brain module for processing arrows! Instead Moore and Corkum (1994) have argued that infants learn that the locus of adults’ social attention predicts the location of significant events and objects, resulting in the establishment of gaze following behaviours. This may be enhanced by the inherent reward value and innate salience of social cues themselves (Triesch et al. 2006). In support of the role of associative learning in the emergence of gaze following, Corkum and Moore (1998) demonstrated that 40 % of infants who were not already gaze following by 8–9 months could be taught to do so when given appropriate feedback. Triesch et al. (2006) have constructed a computational model of the development of gaze following based on social reward-driven learning. According to this account, SR associations might be expected to develop earlier for cues in the environment which have a high social reward value (e.g. faces) compared to more abstract stimuli (e.g. arrows). In line with this account, the earliest age at which arrows have been reported to automatically facilitate attentional shifts is between 3 and 5 years (Ristic et al. 2002). Also consistent with the associative learning account, Guzzon et al. (2010) showed that novel associations can be trained between arbitrary texture pattern cues and direction of peripheral attention, such that the associated patterns presented at fixation exert a similar cueing/congruency effect to that shown by socio-biological cues. Finally, Cole et al. (2015) have shown that gaze cuing effects are not modulated by manipulation of the mental state attribution given to the eyes being viewed (i.e. the effect occurs even if it is clear to the viewer that the actor cannot see the congruent location/object), suggesting that the effect arises due to a direct link between the visual properties of the cue and a learned orienting response rather than being mediated by the social meaning of the cue.

A further prediction of environmental learning accounts of cueing effects is that responses to other socially salient socio-biological cues should also develop early in childhood. Perhaps the most widely used social cue humans use to direct attention is a pointing index finger, and recent studies have demonstrated an apparent automatic orienting of attention elicited by finger pointing which mirrors those of eye gaze cues. In a covert attentional cueing task, targets located in the direction congruent with the direction in which a finger is pointing are identified faster than those appearing in the opposite direction even when the cue is uninformative (Tomonaga and Imura 2009; Ariga and Watanabe 2009; Gregory and Hodgson 2012). Daum et al. (2013) have also shown that 12-month-old babies produce a saccadic orienting response to pointing cues in the absence of motion cues.

To examine the idea that gaze cueing (and cueing effects more generally) reflect acquired associations, rather than innate processing modules, the present study examined the developmental trajectory of different directional cues on the programming of saccadic eye movements in children aged 3–10 years. Saccadic responses in the presence of gaze cues were compared to arrow cues and finger pointing. If eye gaze direction cues are truly special and rely on an innate brain mechanism, then they would be expected to show the greatest effect on young children for whom voluntary inhibitory control mechanisms are not yet fully developed (but for whom innate cue-orienting associations would presumably be fully formed). The effect of other cue types, which are not claimed to rely on innate hard-wired modules, might be expected to take longer to develop and only be present in older children.

## Experiment 1

### Methods

#### Participants

A total of 86 children aged between 3 and 10 years old participated in the study as part of the University of Lincoln’s “Summer Scientist” week, organised specifically for local children and families to take part in fun psychological research studies presented to children as games. Experiments in this programme are set up to help the children and their parents learn more about psychology and the brain, and participate actively in scientific research. Out of the 86 volunteers, we were able to complete eye movement calibration and obtain a full block of trial data in 63 (Mean age = 6.23 years, SD = 1.88). All the children included in the study had either normal or corrected-to-normal vision. Written informed consent was given by the parents of all participants, and ethical approval for this study was granted by the Ethics Committee of the School of Psychology, University of Lincoln. The research was carried out in accordance with the Code of Ethics of the World Medical Association (Declaration of Helsinki).

#### Procedure

The task was presented as a game in which the children had to help the cartoon “Buzzy Bee” by following her with their eyes whilst ignoring the faces, arrows and fingers. At the beginning of the experiment, a three-point calibration procedure was performed with the Buzzy Bee character as the calibration target. An instruction screen was presented before the start of each block which reminded participants to “follow Buzzy Bee and ignore the eyes, arrows and fingers” (along with pictures of example eyes, arrows and finger stimuli), and the experimenter read this out to the child. After the first instruction screen, a block of 12 practice trials was presented, followed by a further calibration, another presentation of the instruction screen followed by the main experimental block of 54 consecutive trials.

**Fig. 1:**
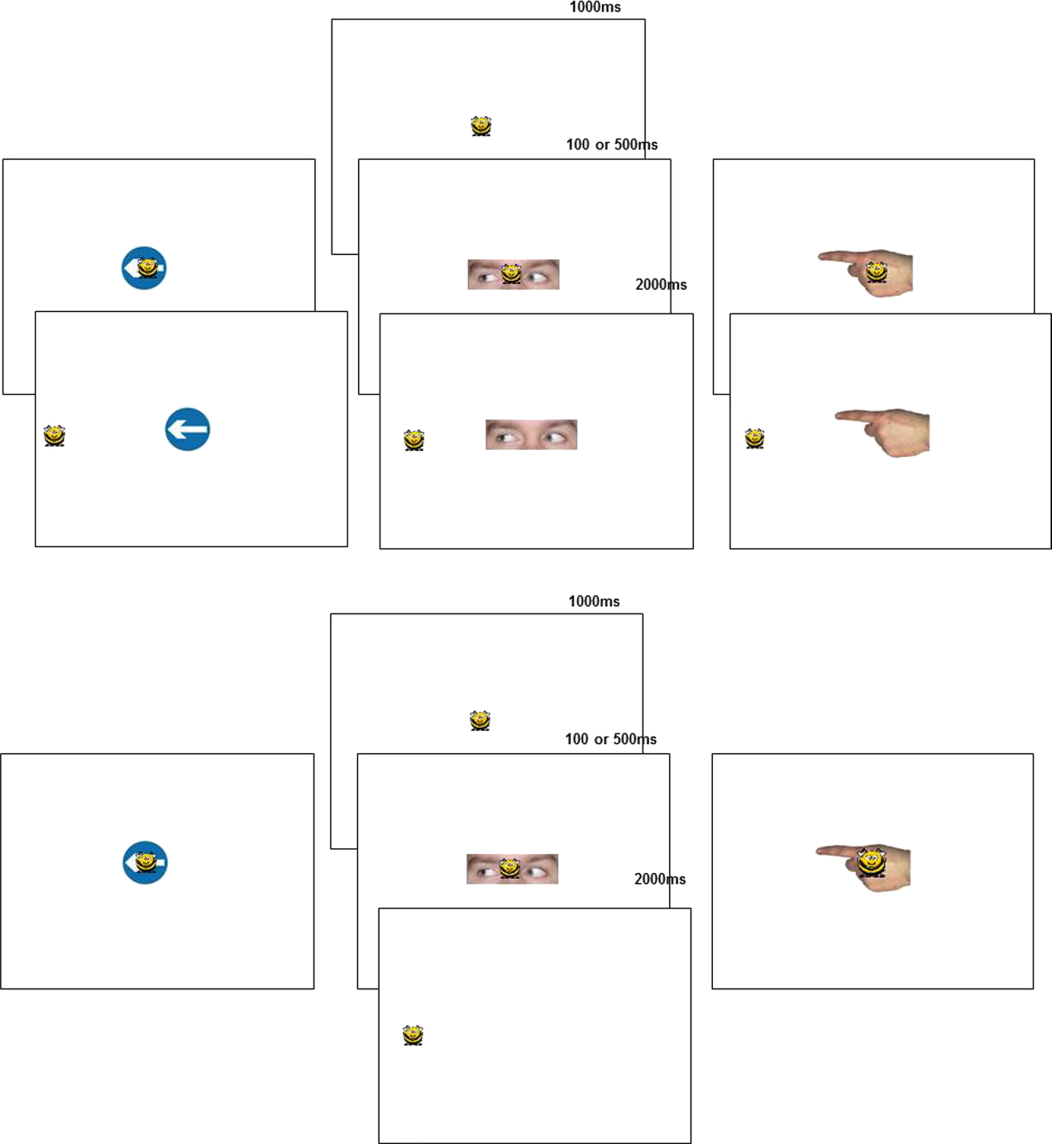
Schematic of the procedure for a congruent gaze cue trial in Experiment 1 (*upper* *panel*) and Experiment 2 (*lower* *panel*). The target (*a* *cartoon* *bee*) was presented at fixation for 1000 ms, after which a cue (either an arrow, eyes or pointing finger) appeared overlapping with the target stimulus at fixation. Following a delay of either 100 or 500 ms, the target stepped to the *left* or *right* for 2000 ms. Children were asked to follow “buzzy bee” using eye movements. The procedure in Experiment 2 was identical to Experiment 1 except that the cue was extinguished simultaneously with the target step to the *left* or *right*

Each trial began with the presentation of Buzzy Bee at central fixation for a duration of 1000 ms (Fig. 1). Following this, a cue was presented which could be pointing left or right and either congruent or incongruent with respect to the target location. After a stimulus onset asynchrony (SOA) of 100 or 500 ms, Buzzy Bee jumped to either the left or right of the screen, where it remained for 2000 ms. The location of the “target” bee was randomised appearing on the left and right of the screen with equal probability and this was read out again by the experimenter. Presentation of trials was randomised across cue type, congruency, SOA, cue direction and target direction. An interval of 1000 ms separated each trial.

### Eye tracking and stimuli

Eye movement recording was carried out by means of the remote desktop mounted EyeLink 1000 system (SR Research, Canada). The system was operated in head free mode and children wore a sticker on their forehead which enabled the eye tracking system to identify pupil and corneal reflection and for head movements to be compensated for whilst maintaining eye tracking. Eye movements were recorded at a frequency of 500 Hz and the reported average accuracy for the setting used is 0.5° of visual angle.

Arrow cue stimuli were based on UK road signs for “keep left” and “keep right” comprising a blue circle with a white arrow subtending 4.45° of visual angle. Gaze cues were cropped colour photographs of a male face, showing just the eye region. The left version of the cue was identical to the right version, but with the sclera and pupil area displayed in mirror image. Finger pointing cues were colour photographs of a male hand with the left version being an exact mirror image of the right pointing finger. The cues subtended 5.52° of visual angle in width. The fixation and target stimulus was a cartoon bee subtending 1° of visual angle. The cues together with the fixation and target stimulus are shown in Fig. 1. Stimuli were presented on a 21″ flat screen ViewSonic LCD monitor set at a refresh rate of 100 Hz and a resolution of 1920 by 1080 pixels. Participants sat approximately 60 cm from the monitor.

**Fig. 2:**
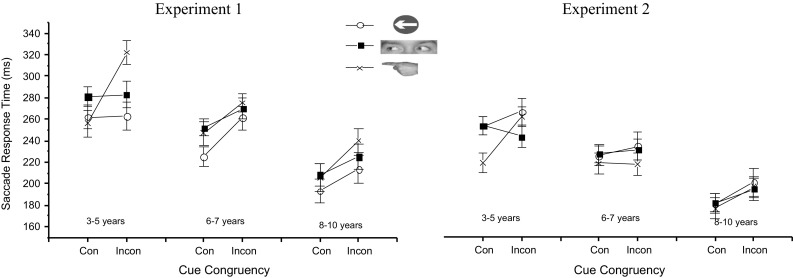
Mean correct SRTs for congruent and incongruent trials over the three age groups for *arrow*, *gaze* and *pointing* *cues* in Experiment 1 (cue–target overlap) and Experiment 2 (simultaneous cue–target offset–onset). *Error* *bars* represent standard error of the mean. *Legend* shows pre-cue stimuli used under each condition

### Data analysis

Data were analysed using SR Research’s Data Viewer software. Saccades were automatically detected by the eye tracker’s software and defined as periods where eye velocity exceeded 30° s^−1^ and an acceleration of 8000° s^−2^. The first saccade detected on each trial following target onset was taken as the primary saccade response for each trial. Only valid saccades directed to the left and right with latencies between 79 and 699 ms and an amplitude of more than 2.0° were included in the analysis (removing anticipatory saccades and excessively slow responses; Fischer et al. 1997).

Participants who responded on at least 1 trial (out of a maximum of 5) per condition and who completed at least 10 experimental trials were entered into the analysis. This procedure left only 36 participants in the main analysis of reaction times, completing 1594 trials between them (see results and discussion for explanation of the high participant “dropout” rate and analysis of response omission errors). Of these remaining trials, those where the first response was initiated in the direction of the target stimulus were considered as correct responses, whilst those made in the opposite direction to Buzzy Bee were classed as errors. Average response times were based on correct responses only.

## Results

### Correct SRT

Figure 2 shows the mean SRTs on congruent and incongruent trials for the three cue types, split across three age groups (3–5 years, 6–7 years and 8–10 years), which were chosen to reflect similar intervals across the age range tested. A four-way mixed measures ANOVA was carried out on the SRT data, with type (arrows, eyes, pointing hand), SOA (100, 800 ms), Congruency (congruent, incongruent) and Age group (3–5 years, 6–7 years and 8–10 years) as factors. There was a main effect of SOA, *F* (1, 33) = 40.17, *p* < .001, *η*_*p*_^2^ = .549, with faster response times at the longer 500 ms SOA (*M* = 238.91 *SE* = 5.11) relative to 100 ms SOA (*M* = 259.32 ms, *SE* = 5.85). There was also a main effect of Congruency, *F* (1, 33) = 80.39, *p* < .001, *η*_*p*_^2^ = .709, with congruent trials producing faster responses (*M* = 235.31 ms, *SE* = 5.28) than incongruent trials (*M* = 260.11 ms, *SE* = 5.70). There was also a main effect of cue type, *F* (2, 66) = 15.09, *p* < .001, *η*_*p*_^2^ = .314. Pairwise comparisons revealed that there was a significant difference after Bonferroni correction between the SRTs on arrow (*M* = 236.50, *SE* = 6.27) and gaze trials (*M* = 253.27, *SE* = 5.25), *p* < .0001, and arrow and pointing trials (*M* = 257.59, *SE* = 5.68), *p* < .0001, but not between gaze and pointing trials, *p* = .252. There was a main effect of age group, *F* (1, 33) = 11.26, *p* < .001, *η*_*p*_^2^ = .406, with SRTs reducing with increasing age. Pairwise comparisons showed that the 3–5-year-olds’ SRTs (*M* = 277.99 ms, *SE* = 9.35) were longer than the 8–10-year-olds’ (*M* = 214.35 ms, *SE* = 9.81), *p* < .001, but not the 6–7-year-olds’ (*M* = 255.01, *SE* = 8.01), *p* = .071. However, the 8–10-year-olds’ SRTs were significantly faster than those of the 6–7-year-olds’, *p* = .003.

**Fig. 3:**
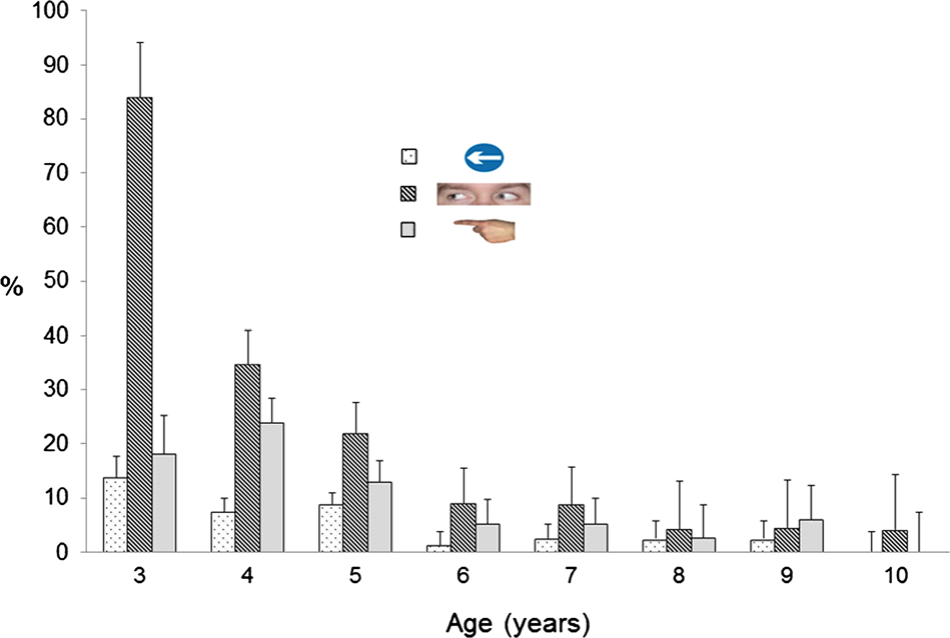
Percentage of omission errors in each cue type across age range

There was a significant interaction between SOA and Age group, *F* (2, 33) = 5.36, *p* = .010, *η*_*p*_^2^ = .245. Bonferroni corrected pairwise comparisons revealed that SRTs at 500 ms SOA were significantly faster than at 100 ms SOA in the 3–5-year-old (*p* = .003) and 6–7-year-old (*p* < .001) groups but not in the 8–10-year-old group (*p* = .140).

There was an additional significant interaction between Cue type and Congruency, *F* (2, 66) = 7.16, *p* = .001, *η*_*p*_^2^ = .178. Bonferroni corrected pairwise comparisons revealed that SRTs on congruent trials were faster than on incongruent trials for all cue types, but that the size of the difference varied across cue types with pointing cues showing the greatest effect (*M* = 42.65 ms, *SE* = 6.46), *p* < .001, followed by arrow cues (*M* = 19.10 ms, *SE* = 3.85), *p* < .001, with the smallest effect in the gaze cue condition (*M* = 12.66, *SE* = 6.00), *p* = .043).

The SOA by Congruency interaction approached significance, *F* (1, 33) = 4.00, *p* = .054, *η*_*p*_^2^ = .108, with a larger congruency effect at 500 ms SOA than at 100 ms SOA. The Cue by SOA, Congruency by Age group and the Cue by Age group interactions were not significant (*p* > .200).

There was a significant three-way interaction between Cue, Congruency and Age group, *F* (4, 66) = 3.75, *p* = .008, *η*_*p*_^2^ = .185 (Fig. 2). Bonferroni corrected comparisons between each condition for this interaction showed that for the youngest group the cue congruency effect was only significant for finger pointing cues (effect of 66 ms ± 11.51 *p* < .001) with gaze cues showing no effect for the youngest age groups and small effects for the older children (gaze cue congruency effect for 6–7-year-olds 18 ms ± 9 *p* = 0.06; 8–10-year-olds 17 ms ± 11 *p* = 0.14).

In summary, pointing cues influenced all age groups, but gaze cues caused small or no significant cueing effects overall. The only cues to significantly influence the 3–5-year-olds’ SRTs were the pointing cues, which had a larger effect than in any of the other groups.

The Cue by SOA by Congruency interaction was also significant, *F* (2, 66) = 3.23, *p* = .046, *η*_*p*_^2^ = .089. Bonferroni corrected pairwise comparisons showed that there was a significant congruency effect for arrows only at the 500 ms SOA (*M* = 34.25 ms, *SE* = 6.45), *p* < .001, for gaze cues at only 100 ms SOA (*M* = 13.79 ms, *SE* = 6.13), *p* = .031 and for pointing cues at both 100 ms (*M* = 34.01 ms, *SE* = 7.84), *p* < .001 and 500 ms SOAs (*M* = 51.28 ms, *SE* = 8.54), *p* < .001.

The Cue by SOA by Age group interaction approached significance, *F* (4, 66) = 2.44, *p* = .055, *η*_*p*_^2^ = .129. The four-way interaction between Age group, Congruency, SOA and Cue was not significant, *F* < 1.20, *p* > .300.

### Correlations

Correlations were also carried out between age (in months) and the cueing effect (incongruent SRT–congruent SRT, in ms) for each cue at each SOA. These analyses revealed a significant negative correlation between age and the cueing effect of the pointing cue at 500 ms SOA, *r* = −.34, *p* = .04, demonstrating that the interfering influence of the pointing cues decreased with age. No other correlations were significant.

### Accuracy

Responses which were initiated in the opposite direction to the peripheral target were classified as errors. Only 33 errors were made, representing 2.07 % of trials. Due to their infrequency, errors were not analysed further.

### Omission errors

It was noted during data collection that some children failed to make any saccade on a proportion of trials. This was particularly noticeable with the youngest children. We therefore investigated the rates of withheld responses in each condition in the experiment. It was due to these omission errors many participants were excluded from the SRT analysis as they did not complete enough trials in each condition (see “Methods” and “Data analysis” sections above). Data from 63 participants were available for the analysis of omission errors as opposed to the 36 participants in the SRT analysis. The proportion of trials where a saccade response was not made was calculated for each participant in each Cue and Congruency condition.

A four-way mixed ANOVA was conducted on these data, with Cue (arrows, eyes, pointing), SOA (100, 500 ms) and Congruency (congruent, incongruent) and Age (3, 4, 5, 6, 7, 8, 9, 10 years) as factors.

The mean rate of omission errors overall was 11.90 %. There was a significant main effect of Cue, *F* (2, 78) = 21.52, *p* < .001, *η*_*p*_^2^ = .356. Post hoc pairwise comparisons demonstrated that there were significantly more omitted responses on eye gaze cue trials (*M* = 21.55 %, *SE* = 2.83) than on either arrow (*M* = 4.92 %, *SE* = 1.08) *p* < .001 or pointing cue trials (*M* = 9.23 %, *SE* = 2.04), *p* < .001 and that there were significantly more omissions on pointing trials than on arrow trials (*p* = .048). There was also a significant main effect of SOA, *F* (1, 39) = 11.20, *p* = .002, *η*_*p*_^2^ = .223, with more responses withheld at the 100 ms SOA (*M* = 14.22 %, *SE* = 1.67), than at the 500 ms SOA (*M* = 9.58 %, *SE* = 1.57). The main effect of Age was also significant, *F* (7, 39) = 7.92, *p* < .001, *η*_*p*_^2^ = .587. The main effect of Congruency was not significant (*F* < 1.00, *p* > .500). There was a significant interaction between Age and Cue, *F* (14, 78) = 4.49, *p* < .001, *η*_*p*_^2^ = .446. The interaction between Age and Cue is shown in Fig. 3. It is clear from the graph that gaze cues were responsible for the majority of omission errors, but this is particularly marked with the 3-year-olds, where there was a disproportionate number of omitted responses with gaze cues (Gaze: *M* = 84.03 %; Pointing: *M* = 18.06 %; Arrows: *M* = 13.89 %). The proportion of responses omission errors with gaze cues decreased with age until 6 years old, where it stabilised.

There was also a significant interaction between SOA and Age, *F* (7, 39) = 2.79, *p* = .019, *η*_*p*_^2^ = .334, which was the result of a particularly large difference between the proportion of missed responses at the 100 ms SOA compared to the 500 ms SOA in the 3-year-old group. None of the other interactions approached significance (*F*_s_ < 1.50, *p*_s_ > .200).

## Discussion

Experiment 1 examined the effect of eye gaze, arrow and finger pointing cues on children’s eye movement responses in a pro-saccade task. The results showed that finger pointing cues, rather than eye gaze cues, had the strongest influence on the youngest children’s saccadic reaction times (SRTs). Overall the magnitude of the effect of finger pointing cues was negatively correlated with age, demonstrating that the influence of pointing cues on saccadic orienting reduced as children got older. In contrast, eye gaze cues had the least influence on children’s SRTs with cueing effects emerging only when data were collapsed across age group. Significant arrow cueing effects were seen in both the 6–7-year-olds and 8–10-year-olds, but there was no such effect in the youngest age group.

Previous studies have reported strong eye gaze cueing effects in children, which attenuate with increasing age (e.g. Ristic et al. 2002). All such studies have used schematic/cartoon faces as gaze cues rather than the photorealistic eye gaze cues used in the present study. This would suggest that young children are less affected by natural eye gaze cues and only show a strong orienting response towards them when they are clearly depicted in schematic form. Other authors have suggested that cueing effects reduce over age due to development of inhibitory mechanism in the frontal cortex which suppress generation of automatic programming of oculomotor responses (Kuhn et al. 2011). The reported correlation of age with effect of finger pointing cues is consistent with this proposal.

Another aspect of attentional control which develops with increasing age is the ability to disengage attention or fixation from a salient stimulus. Consistent with this, on a large number of trials in Experiment 1, children made no sizable saccade at all and remained looking at the centre of the screen throughout the trial. These omission errors were most evident in 3–4-year-old children, with the numbers of omission errors decreasing rapidly with each subsequent year until around 6 years of age. The rate of omitted responses then plateaus and was negligible for children aged 10 and above. Whilst eye gaze cues did not produce cueing effects in young children, they were associated with significantly more missed responses than other cue types.

Although it is interesting, the fact that children found it difficult to disengage fixation from central fixation to fixate the peripheral target makes interpretation of the cueing effects on SRTs problematic. In Experiment 1, the central cue was left visible on the screen after the saccade target moved from the centre to a peripheral position (i.e. fixation “overlap”). This is a problem for interpreting the current results as differences in cueing effects between stimuli and age groups might arise due to the different numbers of missed responses between conditions rather than the directional effect of the cues themselves. Experiment 2 was designed to address this problem using a procedure identical to Experiment 1 with the exception that the central cue offset simultaneously with the peripheral onset of the “Buzzy Bee” target in order to facilitate disengagement of attention and fixation from the central location. In the case of the youngest children, this manipulation was designed to reduce the rate of trials where no response was made for eye gaze cues trials. Any surviving differences in cue congruency effects on SRTs between conditions are less likely to be due to differences in the ability to disengage fixation in younger relative to older children.

## Experiment 2

### Methods

A further 51 children aged between 3 and 10 years old took part in this study as part of the University of Lincoln’s “Summer Scientist” week the following year of which 33 completed the eye tracker calibration and a full experimental block as defined in Experiment 1, completing a total of 843 trials between them (Mean age = 6.31 years, *SD* = 2.16). All participants had either normal or corrected-to-normal vision. Written informed consent was given by the parents of all participants, and ethical approval for this experiment was granted by the Ethics Committee of the School of Psychology, University of Lincoln. The research was carried out in accordance with the Code of Ethics of the World Medical Association (Declaration of Helsinki).

The design and materials for Experiment 2 were identical to Experiment 1. The procedure was the same as in Experiment 1, except that the cue stimulus was removed from the display simultaneously with the target appearance (Fig. 1).

### Results

#### Omission errors

As predicted, the rates of omitted responses were far lower than in Experiment 1. In Experiment 2, participants failed to make a saccade on only 15 trials, representing 0.9 % of trials overall. Due to the small number of omission errors, they were not statistically analysed.

#### Correct SRT

Figure 2 shows the mean correct SRTs across conditions. A three-way mixed ANOVA was performed on these SRTs, with Cue type (arrows, gaze, pointing), Congruency (congruent, incongruent) and Age group (3–5 years, 6–7 years and 8–10 years) as factors. There was a main effect of Congruency, *F* (1, 30) = 8.75, *p* = .006, *η*_*p*_^2^ = .226, with faster responses overall on congruent trials (*M* = 214.90 ms, *SE* = 3.78) than incongruent trials (*M* = 226.97 ms, *SE* = 4.70). There was also a main effect of Cue type, *F* (2, 60) = 3.29, *p* = .044, *η*_*p*_^2^ = .099. Pairwise comparisons showed that responses in the presence of pointing cues were significantly faster than those made in the presence of arrows (*p* = .016), but not gaze (*p* = .291), with no difference between the SRTs made on gaze and arrow trials (*p* = .156). There was also a main effect of Age group, *F* (1, 30) = 23.53, *p* < .001, *η*_*p*_^2^ = .611, with SRTs reducing as age increased.

Although the Cue by Congruency by Age group interaction did not reach significance, *p* = .225, Bonferroni corrected pairwise comparisons were conducted to assess the significance of congruency effects across age groups and cue types to examine whether the same pattern of difference in cueing across age observed in Experiment 1 was also evident in Experiment 2. The only significant cueing effect was found for pointing cues in the 3–5-year-old group, (*M* = 42.17 ms, *SE* = 10.53), *p* < .001. The pointing cueing effect approached significance in the 8–10-year-old group, (*M* = 20.69 ms, *SE* = 11.00), *p* = .070, but none of the other congruency comparisons approached significance, *p*s > .230. None of the other interactions reached significance.

#### Correlations

Bivariate Pearson’s correlations were computed between the mean cueing effect for each cue type per participant and exact age. The only correlation to reach significance was between the gaze cueing effect and age, *r* = .367, *p* = .035, with the gaze cue’s effect increasing with age. For all other correlations, *p* > .375.

#### Accuracy

Participants made only 38 errors in total, representing 3.66 % of trials and this was considered too little data to be analysed statistically.

## Discussion

Experiment 2 aimed to replicate the findings of Experiment 1 under conditions where the central cue was removed from the screen simultaneously with the peripheral target onset rather than overlapping with target shifts to the left/right as in Experiment 1. As expected, extinguishing the cue stimulus had the effect of dramatically reducing the number of trials where fixation was maintained at the central location. In terms of the effect of cue congruency on SRTs, however, Experiment 2 supported and extended the findings of Experiment 1. Finger pointing cues showed a strong cue congruency effect on saccade responses in 3–5-year-old children. As found for Experiment 1, however, gaze direction cues had very little effect on SRTs.

As the two experiments were closely matched other than with regard to the timing of the cue offset, we carried out a cross-experiment analysis on the data collapsed across the two experiments. This cross-experiment analysis^1^ demonstrated that overall, SRTs were significantly faster in Experiment 2 relative to Experiment 1, indicating that eye movement initiation was facilitated by the offset of the central cues. The cross-experiment analysis also confirmed that pointing cues consistently produced the largest cueing effects across age groups whilst gaze cues caused the smallest as well as confirming the presence of a consistent three-way interaction between Age, Cue type and Congruency.

The increase in SRTs observed under fixation overlap conditions (Experiment 1) is consistent with the well-known fixation overlap effect on eye movements (e.g. Saslow 1967). Other findings suggest that this facilitatory effect of fixation offset is not only caused by more rapid attentional disengagement from fixation, but also due to a “warning signal” effect of fixation offsets in predicting the timing of onset of the peripheral stimulus (Reuter-Lorenz et al. 1991; Reuter-Lorenz et al. 1995; Ross and Ross 1980). It is conceivable that younger children might be less able to utilise the predictive nature of fixation offset in this way, although the current data do not offer strong support for this possibility as SRTs for the different age groups became more similar under fixation offset conditions (Experiment 2). Instead, the interaction between Age, Cue type and Congruency suggests that younger children find it more difficult that older children to disengage fixation and this “sticky fixation” effect is most marked for eye gaze cues.

## General discussion

In two experiments, we examined the influence of gaze, arrow and hand pointing cues on saccadic responses to a peripheral target in 3–10-year-old children in order to examine the development of cue congruency effects on SRTs. The results are the first to demonstrate dissociable influences of pointing, gaze and arrow cues on saccadic orienting between ages 3 and 10 years and indicate that pointing fingers rather than averted eye gaze are the most effective stimuli for eliciting shifts in visual attention in young children.

We originally set out to examine two alternative explanations for commonly observed eye gaze cuing effects on covert visual attention and eye movement programming. Firstly, it has been proposed that processing eye gaze direction relies on an innate or a “hard-wired” eye gaze direction module in the brain (Baron-Cohen 1995). The alternative view is that associations are learned from the environment which link perceptual cues with the direction of objects or locations of interest, such that over time relevant stimuli become associated with directional shifts in visuospatial attention (Moore and Corkum 1994; Corkum and Moore 1998; Triesch et al. 2006; Cole et al. 2015). Within this latter account eye gaze direction is not innately associated with attentional shifts, but is one of a number of biological and non-biological cues in the environment which can be subject to associative learning processes.

A positive correlation was found between the size of eye gaze cueing effects and age in Experiment 2. This is not consistent with the existence of the innate eye gaze direction mechanism as an explanation for eye gaze cueing effects as has been proposed elsewhere (Baron-Cohen et al. 1995; Hood et al. 1998), as this would predict either a stable gaze cueing effect with increasing age or a decreasing effect, due to development of cognitive inhibitory control mechanisms. At the same time eye gaze cues were clearly highly salient stimuli for the younger children to the extent that they found it hard to disengage fixation from them when they overlapped with the peripheral target onset (Experiment 1). As experimenters watching the children perform the task, we found this effect to be reminiscent of target extinction phenomena seen in adult patients with hemi-spatial neglect (Losier and Klein 2001). Oculomotor engagement/disengagement is thought to be related to increases/decreases in activity of fixation-related neurons in the superior colliculus which are modified by fixation offset and attentional factors (Dorris and Munoz 1995; Dorris et al. 1997). The strong engagement effect elicited by gaze cues in our youngest children suggests an enhanced attentional response to eye gaze cues in young children, and recent studies using event-related potentials have also suggested this may be the case. Taylor et al. (2001) found that 4–5-year-old children showed very early N170 waveform response to eyes when compared to older children, suggesting that eyes constitute a particularly salient and engaging stimulus for this age group.

In agreement with a large body of past studies in adults, our study found similar cueing effects for gaze and arrow cues (e.g. Tipples 2002, 2008; Kuhn and Kingstone 2009; Hermens and Walker 2010). Whilst early studies reported gaze cues to have a more pronounced influence than arrows, subsequent investigations have demonstrated that these two directional cues have almost indistinguishable effects on eye movements and covert attention in a range of tasks (Tipples 2002; Friesen et al. 2004; Quadflieg et al. 2004; Kuhn and Benson 2007; Tipples 2008; Kuhn and Kingstone 2009; Hermens and Walker 2010; Galfano et al. 2012). This suggests that in adults the mechanism by which these stimuli operate on attention is likely to be the same. At the neural level, orienting to gaze and arrows is known to recruit similar brain networks (Callejas et al. 2014) with some differentiation related to their semantic and contextual differences (Engell et al. 2010; Marotta et al. 2012). However, our results showed that arrow cueing effects do not emerge until approximately 6 years of age. By age 6, children would have been sufficiently exposed to arrows in their environment for learned SR associations to develop.

An interesting and unexpected aspect of our results was that an image of a pointing index finger exerts a particularly strong influence on saccadic eye movement programming in young children. Other work has shown that pointing is a socially salient cue for young children (e.g. Butterworth and Itakura 2000; Carpenter et al. 1998; Deák et al. 2000; Matthews et al. 2012; Morissette et al. 1995) even more so than gaze direction (Butterworth and Itakura 2000; Deák et al. 2008). One possibility is that young infants learn to associate the outstretched hand of an adult with the vicinity of interesting events at an earlier stage than other cues, due simply to the fact that adult hands are more salient in their environment as they tend to occur lower in the visual field than the eyes/faces of adults they interact with. The effect was found to reduce with increasing age, consistent with maturation of prefrontal cortical circuitry mediating inhibitory control over automatic responding to finger pointing cues in older children.

The evidence that very young children make rapid shifts in overt or covert attention in response to eye gaze cues is limited with some work indicating that apparent motion plays a key role in apparent gaze cueing effects in babies (Hood et al. 1998; Farroni et al. 2000). Our work also argues against a “hard-wired” mechanism established early in brain development, but this does not necessarily exclude the possibility that some aspects of orienting to social biological cues might arise from innate biases in attention. Contemporary theories of development suggest that behaviours may be encoded in an individual’s genetic makeup even if not observable at birth, only emerging later in development (Stiles 2011). Contemporary perspectives on the nature versus nurture dichotomy try to understand how inherited traits interact with the environment to produce behaviour (Rutter 2006; Sameroff 2010). Such a biopsychosocial approach is likely to be particularly pertinent to understanding a social cognitive process like gaze following. Our data support other work which demonstrates that young children appear to have an innate preference for faces above non-social objects (e.g. Batki et al. 2000; Grossmann et al. 2008) as the youngest participants in Experiment 1 had difficulty in disengaging their attention from the eye gaze cues presented at fixation. The development of gaze following behaviour and gaze cueing effects may be facilitated by this predisposition to attend to faces coupled with the development of stimulus–response associations reinforced by social reward value (Corkum and Moore 1998; Triesch et al. 2006; Cole et al. 2015).

Taken together, the results are consistent with an account which combines the concept of innate salience for eye gaze with the flexible learning of SR associations through environmental experience and the progressive maturation of volitional attentional control circuits in the frontal cortex. Eyes/faces (irrespective of the direction they point) are salient stimuli for capturing attention in early life, and this is evidenced by the high rate of omission errors on eye gaze cue trials even in our youngest participants. However, discriminating the direction of gaze, disengaging attention from the central stimulus and transforming another’s eye gaze direction into the coordinates of a corresponding egocentric location rely on more complex mechanisms that take longer to develop. Thus, whilst young children may show a strong tendency to fixate/overengage attention on centrally presented eye gaze stimuli, it is only later that they acquire the ability to voluntarily disengage their own attention from another’s eyes and shift attention towards the location indicated by another’s point of gaze.

The reduction in the size of the finger pointing cue congruency effect with increasing age is also consistent with the development of volitional inhibitory control centres in the prefrontal cortex (Luna et al. 2008), as once strong cue–location associations have been established they would need to be inhibited in order to execute saccades towards cue incongruent locations. However, another factor which may explain the reduction in pointing cues’ effects with increasing age relates to the relationship between pointing and language. For example, the use of pointing appears to predict later language acquisition (Tomasello et al. 2007) and periods of intense language development appear to disrupt the use of non-verbal communication cues such as pointing (Iverson and Goldin-Meadow 2005). The influence of pointing finger cues on attention systems may naturally reduce as children become less reliant on non-verbal communication and language skills develop.

## Conclusion

The results of the present study suggest that the effect of eye gaze and finger pointing cues on programming of eye movements develop along different trajectories in children under the age of 10 years. Contrary to previous research, we found little evidence to support an innate eye gaze direction orienting mechanism. Eye gaze cues were found to be least effective of the cues tested in influencing children’s saccadic responses. Conversely, a large finger pointing cue congruency effect was evident in the youngest age group, which decreased in magnitude with increasing years. We suggest that the developmental trajectory of cue congruency effects across the different cues reported here and elsewhere is best explained through the existence of a general stimulus–response associative learning mechanism which can capture probabilistic associations between environmental cues and spatial locations of interest which are repeatedly paired with them. An adult’s pointing finger may be among the earliest cues within a child’s perceptual environment which acquires directional associations of this type.

## Footnotes

1 Results of the cross-experiment SRT analysis: significant effects of experiment, *F* (1, 63) = 18.58, *p* < .001, *η*_*p*_^2^ = .228, Age group, *F* (2, 63) = 29.91, *p* < .001, *η*_*p*_^2^ = .487, Congruency, *F* (1, 63) = 57.49, *p* < .001, *η*_*p*_^2^ = . 477 interactions between: Congruency × Experiment, *F* (1, 63) = 14.82, *p* < .001, *η*_*p*_^2^ = .190, Cue × Congruency, *F* (2, 126) = 6.78, *p* = .002, *η*_*p*_^2^ = .097 Cue type, Congruency and Age group, *F* (2, 126) = 4.30, *p* = .002, *η*_*p*_^2^ = .120.
